# Rice straw-derived cellulose: a comparative study of various pre-treatment technologies and its conversion to nanofibres

**DOI:** 10.1038/s41598-023-43535-7

**Published:** 2023-09-28

**Authors:** Neha Sharma, Benjamin James Allardyce, Rangam Rajkhowa, Ruchi Agrawal

**Affiliations:** 1 TERI Deakin Nanobiotechnology Centre, TERI Gram, Gual Pahari, Gurugram, India; 2 Institute for Frontier Materials, Deakin University, Geelong, Australia

**Keywords:** Environmental impact, Nanoscale materials

## Abstract

Rice straw is a waste product generated after the harvesting of rice crops and is commonly disposed of by burning it off in open fields. This study explored the potential for the extraction and conversion of cellulose to cellulose nanofibres (CNFs) to be used as smart delivery systems for fertilizers applications. In this study, alkali, steam explosion, and organosolv treatments were investigated for cellulose extraction efficiency. The morphological characterization of cellulose showed smooth fibrillar structures. Fourier transform infrared spectroscopy represented significant removal of non-cellulosic components in treatments. The crystallinity increased from 52.2 to 65% in CNFs after fibrillation. Cellulose nanofibres (CNFs) had an average diameter of 37.4 nm and − 25.2 mV surface charges as determined by SEM and zeta potential, respectively, which have desired properties for holding fertilizers. Therefore, this study paves the way for value-added uses of rice straw as alternatives to current environmentally harmful practices.

## Introduction

Among the world's three most common staple grains, rice holds a central position in feeding almost half of the world's population and meeting the energy needs of the masses. For several nations, it is also important for earning foreign exchange, which directly impacts their economies^1^. Rice straw production at the global level now amounts to 731 million metric tonnes, with a distribution of 1.7, 3.9, 20.9, 37.2 and 667.6 million metric tonnes in Oceania, Europe, Africa, America, and Asia, respectively^2–5^. Presently, there is a substantial issue with handling this vast amount of biomass, which poses a serious threat to the Indian economy and ecology^6^. Due to modern agriculture, which uses a multi-cropping system where the fields must be left vacant for a limited time period (20–25 days) before a subsequent crop can be sown^7^. Hence, due to the lack of economically viable alternatives to using straw, Indian rice farmers, especially in Punjab and Haryana in the north, prefer to burn straw in their fields. Almost 178 metric tonnes of crop residues are burned in Indian farms, 87 metric tonnes of which is rice straw. Even more surprising, the practise of burning rice straw is rapidly spreading in East Indian states such as West Bengal, Orissa, Bihar, and Jharkhand^8^.

**Figure 1:**
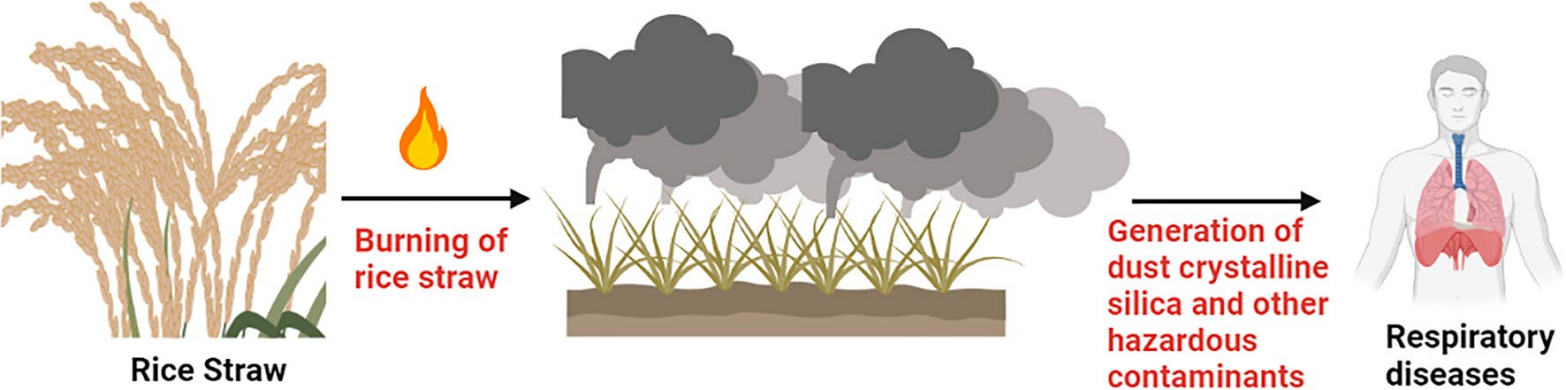
Insight showing impact of burning rice straw.

The open burning of rice straw has harmful environmental impacts (Fig. 1). A recent report of the UNFCC shared astonishing data showing that 40–60% of anthropogenic emissions were contributed by agricultural burning in Southeast Asia^9^. First and foremost, burning causes emissions of detrimental gases and particulate matter, which significantly increase air pollution and greenhouse gas and carbon footprints. In the statistical dataset of greenhouse gas (GHG) emissions, approximately 0.7–4.1 g of methane and 0.019–0.057 g of nitrous oxide are generated from the burning of per kg of dry rice straw. Moreover, other gaseous pollutants, viz., carbon dioxide, sulphur dioxide, volatile organic compounds, and carcinogenic polycyclic aromatic hydrocarbons (PAH), are also produced^10^. In addition, burning rice straw is also an essential source for aerosol particles (coarse dust particles and fine particles), which affect the air quality and reduce visibility^11^. Rice straw burning also poses a threat to the ecosystem since it depletes soil silicon and potassium stocks by losing significant amounts of important nutrients, including nitrogen (5.5 kg), phosphorous (25 kg), and sulphur (1.2 kg). This problem is prevalent in India, Bangladesh, and Nepal^12^.

Rice straw-derived cellulose can be used as a potent raw material for hydrogel, composite, or carrier material for slow-release fertilizer thereby assisting in lowering GHG (greenhouse gas) emissions^1^. CNF is commercially produced from softwood and hardwood pulps via grinding, homogenization, and ball milling processes^13^. The grinding and homogenization processes may result in fragile fibres and involve high energy costs; thus, ball milling is an environmentally friendly and economical process. Nanofibers are used because of their non-toxic nature, high aspect ratio, excellent mechanical attributes, and extensive hydrogen bonding capacity^14^. A study by Uddin et al. used a top-down approach to create silk nanofibres from degummed *Bombyx mori* silk fibres using a sequential process of cutter milling, agitated bead milling, heating, and high-pressure homogenization that tends to produce 200–300 nm fibres in diameter^15^.

Cellulose nanofibres are poised to play a vital role in the advancement of a bio-based economy due to their diversity in application and nearly limitless supplies of renewable feedstocks^16^. Therefore, it is crucial to investigate the cellulose derived from rice straw on the basis of chemical and morphological parameters for its best possible uses. In the present study, rice straw was pre-treated through alkali, steam explosion, and organosolv methods to isolate cellulose and further converted to CNF through ballmilling, followed by characterization to identify their morphology (SEM, TEM), functional groups (FTIR), and thermal features. Thus, cellulose nanofibres can be efficiently used in agricultural applications. The novelty of the study is to extract cellulose from various processes and to determine the efficiency of different methods to develop value–added products from agro-waste.

## Materials and methods

### Biomass collection, preparation, and storage

Rice straw (*Oryza sativa*) was collected in August 2020 from the fields of Mathura District (with a latitude of 27.49° N, and a longitude of 77.67° E), Uttar Pradesh, India. Further, it was air dried, shredded into a size of 5 mm with a knife mill, and stored in an airtight, sterilized container for future investigations.

### Extraction of cellulose-rich variants from rice straw using different pulping methods

The different pulping processes used for the pre-treatment of rice straw to eliminate the non-cellulosic components from the raw material are as follows:

### Alkali-based pulping with NaOH

The alkali-based pulping method is efficient in the removal of non-cellulosic materials such as hemicellulose, lignin, pectin, waxes, and other impurities^17^. Around 30 g of rice straw was weighed and mixed with NaOH solutions of various concentrations (2%, 5%, 8%, and 12%) to make up the volume of 300 ml (w/v). The solution was mixed and heated at 121 °C under a pressure of 1 bar for 1 h in a high pressure reactor. The solution was allowed to cool and then centrifuged at 5000 rpm for 10 min to remove the soluble lignin. The solid residue was allowed to dry overnight at 60 °C. Further, the insoluble fraction was bleached with a 5% (w/v) solution of sodium chlorite in 1 M glacial acetic acid at a solid loading of 10% (w/w). The solution was mixed and kept in the water bath at 70 °C for 10 min^18^. The pulping experiment was performed in triplicate to minimize the handling errors that occurred during the washing process.

### Steam explosion pre-treatment

Steam pre-treatment is a green process as it utilizes steam for the rupturing of the outer pectin layer of the lignocellulosic cell wall. Around 400 g of rice straw was treated in a high-pressure reactor (Nano-Mag Technologies Private Limited, Mumbai, India) under high-pressure steam of 10–15 bar for 10 min and at an operating temperature of 180 °C as previously mentioned^19^. After the steam explosion, the cellulose-rich residue was recovered and stored for future studies.

### Organsolvent treatment

The organosolvent pre-treatment process is another green process as it involves the utilization of organic solvents such as ethanol for the delignification of lignocellulosic material^20^. Rice straw was treated with formic acid and ethanol (ratio 3:1) at 10% solid loading. The reaction was carried out in a high-pressure reactor (Nano-Mag Technologies Private Limited, Mumbai, India) at 160 °C for 1 h. Then, the residue was collected, dried, and stored for further analysis.

### Cellulose nanofibrillation through ball milling process

**Figure 2:**
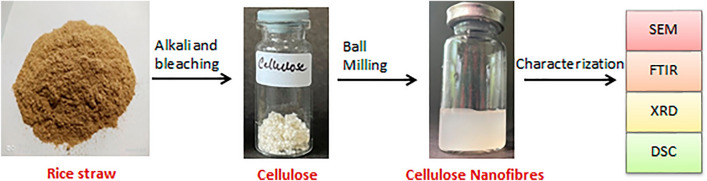
Process flow of extraction of cellulose from rice straw and production of cellulose nanofibres.

Cellulose nanofibres were produced using a bench-top planetary mill (Nano Science & Technology Company (NST), NST 0.4L, Korea). Around 3% (w/v) solution of the extracted cellulose was subjected to milling at room temperature using stainless steel balls (diameter 5 mm) at a ball-to-material ratio of 80:1 and a speed of 300 rpm for periods of 3 h^21^. After milling, the balls were separated, and the material was collected for future characterization (Fig. 2).

### Characterization of cellulose and cellulose nanofibres (CNFs)

#### Compositional analysis

Rice straw is primarily composed of cellulose, hemicellulose, and lignin. The main constituent of hemicellulose is xylan, with a small amount of arabinose. Additionally, other sugars that make up the structural components are galactan, arabinan, mannan, and acid-soluble lignin. On the contrary, the non-structural components are estimated as proteins and extractives^22^. Conceptually, the carbohydrate amount will increase after the efficient delignification process because the carbohydrates, cellulose and hemicellulose, can be released from the bonded lignin walls^23^. The structural carbohydrates and lignin were characterized through the standard laboratory analytical protocol of the National Renewable Energy Laboratory (NREL LAP)^24^. Before analysis, the moisture content of samples was recorded using a moisture analyzer (Scaletec ATS 210), which should be less than 10%. Around 300 mg of rice straw were hydrolyzed with 72% sulfuric acid for 1 h at 25 °C on a magnetic stirrer and further autoclaved at 121 °C for another h. The hydrolyzed samples were filtered through a vacuum filter to analyze the insoluble lignin content, while the hydrolysate was used to determine the sugar content through HPLC (Shimadzu) equipped with a Rezex monosaccharide H + column (100 × 7.8 mm) and refractive index (RI) detector. The mobile phase was 0.005 M H_2_SO_4_. The estimations were done at a flow rate of 0.6 ml/min with a sample injection volume of 20 µl. The operating temperature of the oven was set at 40 °C. The sugar standards (cellobiose, glucose, xylose, arabinose, acetic acid, and ethanol) were determined through an RI detector, while hydroxymethylfurfural (HMF) and furfural standards were estimated using a PDA detector. The contents of acid-soluble lignin were also determined in the hydrolysate using the UV–Vis spectrophotometer at a wavelength of 205 nm.

### Fourier transform infrared spectroscopy (FTIR)

The rice straw and extracted cellulose samples were characterized through Fourier transform infrared spectroscopy (Nicolet, 6700) with a scanning wavenumber of 4000–400 cm^−1^ for the identification of functional groups. The resolution was set at 4 cm^−1^ with 128 scans per spectrum^25^.

### Scanning electron microscopy (SEM)

The morphology of rice straw and extracted cellulose samples was determined through scanning electron microscopy (Zeiss EVO MA 10). The sample was prepared by dispersing the samples in 70% (v/v) ethanol and dropping them on a metal stub covered with aluminium tape. The sample was dried using pressurized air, sputter-coated with a 12–15 nm gold layer using a mini sputter coater (SC7620, Quorum Technologies) under vacuum, and examined under a scanning electron microscope for changes in the morphology and fibrillar structure after treatment^26^.

### Transmission electron microscopy (TEM)

TEM was performed to further study the structural modifications in the cellulose variants after pre-treatment. The diluted sample (0.01% w/w) was coated on a copper grid, followed by air drying and imaging using a transmission electron microscope (TECNAI G2 T20 TWIN), operated at 100 kV.

### Thermal analysis through differential scanning calorimetry (DSC)

The thermal characterization of rice straw and cellulose was performed through differential scanning calorimetry (DSC, TA Q200) to determine the thermal stability of the cellulose and cellulose nanofibres samples. Around 6 mg of the sample was pelleted in the standard aluminium pan. Each sample was heated from room temperature to 400 °C at a rate of 5 °C/min under nitrogen gas and analyzed^27^.

### X-ray diffraction analysis

The XRD was used to analyse the phase composition of samples using Miniflex XRD with Cu-Kα radiation at 30 mA and 40 kV. The dried sample was placed onto a glass sample holder (0.5 mm), and the smear was formed with another glass slide. The sample was scanned between 5° and 50° with a scan rate of 2°/min. The crystallinity index (CrI) of the cellulose was determined to investigate its microstructure and mechanical properties. It is determined through X-ray diffraction analysis which is calculated from Eq. (1):

1 $$CrI(\%)=\left(I002-Iam\right)\div I002\times 100$$

where I002 is the maximum diffraction intensity, located around 2θ = 22.5° and corresponds to the crystalline region; Iam is the minimum diffraction intensity, located around 2θ = 18° and corresponds to the amorphous material^28^.

### Zeta potential

Zeta potential is used to measure the stability and surface charge of the suspension. The cellulose nanofibres solution of 0.03% w/v was prepared and sonicated to make the solution homogenous. Around 1 ml of a sonicated solution of CNF was transferred into a polystyrene macro-cuvette. Zeta potential was measured using a dynamic light scattering instrument (DLS, Malvern instrument, Zen 3690, UK). The measurements were taken in triplicate^29^.

### Statistical analysis

All the experiments were performed in triplicate. The data points presented are the average of the triplicates and have been indicated in all the figures along with the standard deviation.

### Research involving plant material

The MS involves the utilization of agro-waste (rice straw) generated from rice in the present study, which complies with international, national, and/or institutional guidelines. The authors are trying to develop a value-added product from the agro-waste that does not pose any threat to the plant species. The institute and government are encouraging the utilization of waste for value-added products and providing funds for innovative approaches.

## Results and discussions

Rice straw has a heterogeneous biochemical structure and is very diverse^30^. Therefore, it should be characterized based on both its physical and chemical structural properties before and after the pre-treatment.

### Compositional and yield analysis of the rice straw and extracted cellulose variants

The pre-treatment process facilitates the removal of non-cellulosic components from the raw material. In this work, three common pre-treatment technologies (alkali, steam, and organosolvent) have been compared based on the cellulose yield and analytical characterization of the cellulose variants. In alkaline conditions, lignin is removed, while in acidic conditions, hemicellulose (an amorphous structure) is removed from the fibres^31^. The efficiency of each pre-treatment would be determined by correlating the recovery or yield of the fraction with the content of the cellulose in the fraction. For this, compositional analysis was performed using the NREL LAP method (NREL, LAP, USA). The recovery of extracted cellulose after different pre-treatment methods has been calculated using the following formula:

$$Yield \left(\%\right)=Final \; weight \; \left(g\right)\div Initial \; weight \; (g)\times 100$$

**Figure 3:**
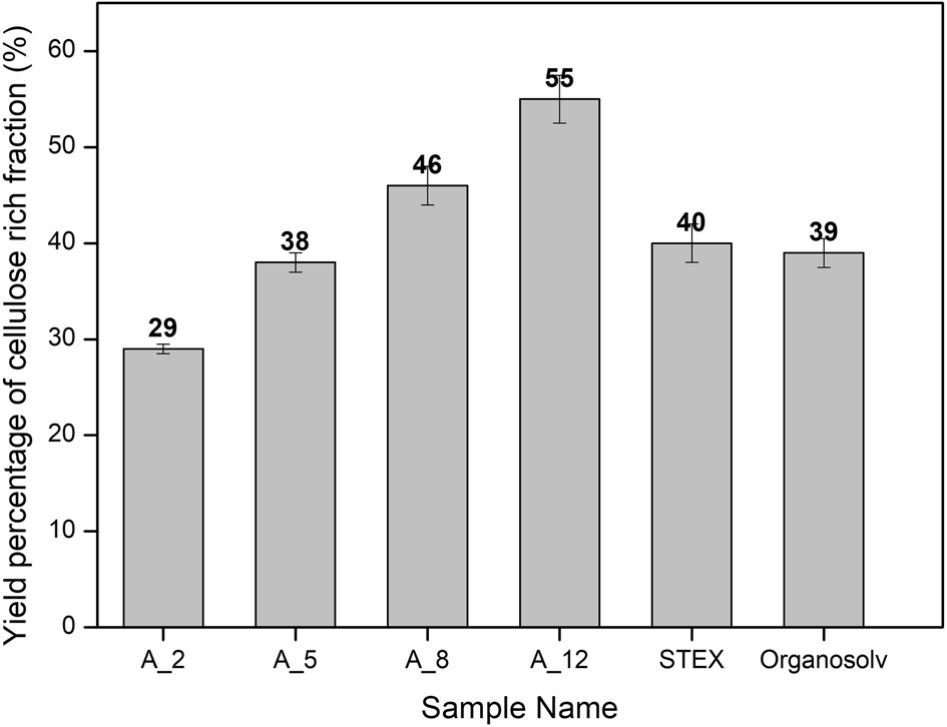
Recovery of cellulose-rich residue (Yield%) as determined after various pre-treatment methods.

The highest amount of cellulose yield (55%) was obtained in the case of the A_12 treatment, where 12% of alkali was used, as mentioned in Table 1. Further, the yield percentage is represented in Fig. 3.

**Table 1:** Proximate composition of rice straw and extracted cellulose as determined by NREL LAP.

S. no.	Sample name	Chemical composition (wt%)				Yield (%)
		α-Cellulose	Hemicellulose	Lignin	Moisture	
1	Rice straw	39.2 ± 0.1	20.5 ± 0.05	21.1 ± 0.1	8.4 ± 0.01	–
2	A_2	51.6 ± 0.05	12.6 ± 0.01	11.7 ± 0.01	7.8 ± 0.05	29
3	A_5	67.8 ± 0.2	12.5 ± 0.02	9.1 ± 0.5	7.3 ± 0.1	38
4	A_8	71.3 ± 0.1	13.4 ± 0.01	6.2 ± 0.1	7.2 ± 0.04	46
5	A_12	85.5 ± 0.1	6.2 ± 0.03	2.3 ± 0.01	7.0 ± 0.01	55
6	STEX	52.7 ± 0.1	6.8 ± 0.02	20.3 ± 0.1	8.4 ± 0.05	40
7	Organosolv	46.9 ± 0.05	13.7 ± 0.3	16.3 ± 0.2	9.4 ± 0.1	39

A_2, A_5, A_8, and A_12 refer to cellulose samples obtained with different concentrations of alkali NaOH (2–12%), while STEX and OS refer to cellulose samples obtained via steam explosion treatment and organosolvent treatment, respectively.

In this study, it was shown that α-cellulose content increased when the rice straw was pretreated with different concentrations of NaOH, and maximum cellulose content was observed in A_12 (85.5%) (Table 1). A report by Senthilkumar et al. represented that the agro-waste can be used as an alternative source of cellulose, and in their investigation, delignification was carried out using 12% alkali with a significant recovery of cellulose^32,33^. STEX and organosolv pre-treatment showed 52.7 and 46.9% cellulose content, respectively. Steam explosion pre-treatment causes explosive decompression and disintegration of the rice straw cell wall structure and the conversion of the polymeric matrix into finer components^34^.

### Morphological characterization of rice straw and extracted cellulose by SEM and TEM

**Figure 4:**
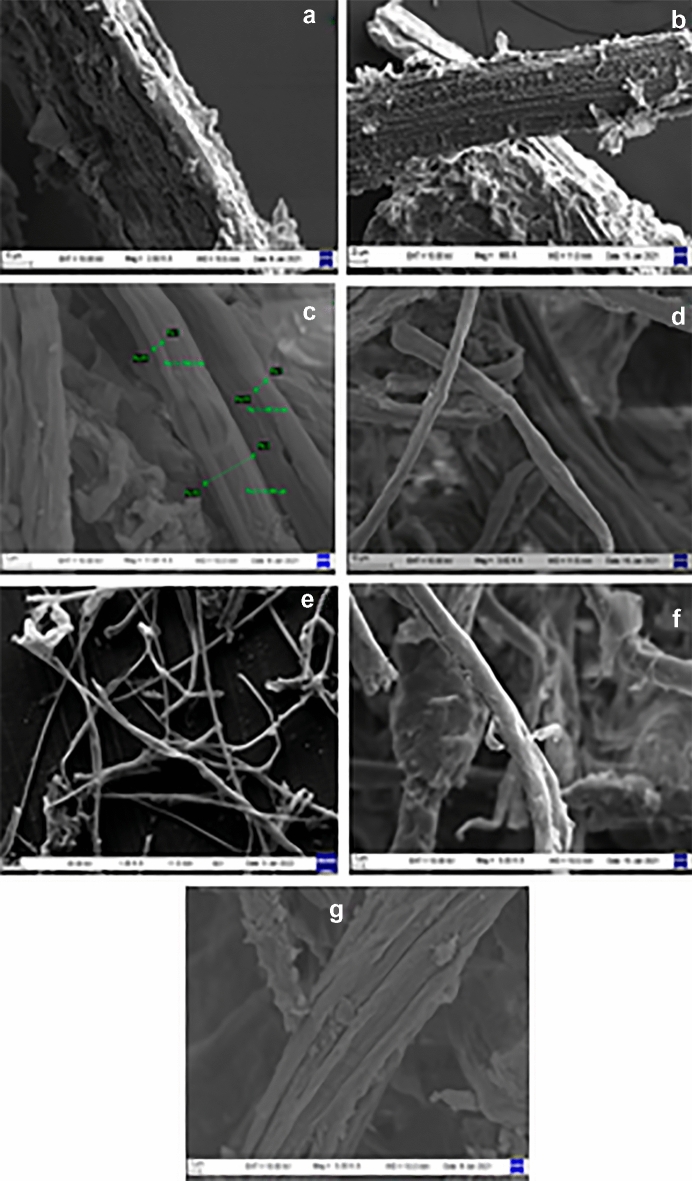
Scanning Electron Microscopy (SEM) images of treated samples: (**a**) rice straw, (**b**) A_2, (**c**) A_5, (**d**) A_8, (**e**) A_12, (**f**) organosolv and (**g**) steam explosion.

The rice straw and extracted cellulose samples have been visualized under a scanning electron microscope to identify the morphological variations after treatment. The scanning electron micrographs of the rice straw indicated a compact and intact structure with a rough surface, which could be due to the presence of non-cellulosic components (Fig. 4a). A similar report by Wang et al. showed the spike-like structure of the husk, which was reported to be formed due to the cementing of non-cellulosic material combined with the celluloses of the fibres and is accountable for the rigidity, impermeability, and protection of the entrenched cellulose framework^35^. A similar micrograph was seen in the A_2 sample, where fibrils were seen intact as in the native rice straw because a lower concentration of alkali (2%) was unable to delignify the fibres efficiently and the lignin chain was not broken down (Fig. 4b). The loosening of the fibrillar structure and appearance or disappearance of a smooth surface were observed in the alkali-treated samples (A_5, A_8, and A_12), which signify the removal of non-cellulosic components and delignification (Fig. 4c–e)^36^. The morphology of organosolv-treated cellulose also indicated effective changes when compared with the native rice straw. A similar observation was made that reported the disappearance of pectin channels on the surface of organosolv-treated rice straw, indicating a loosening of the structure and the removal of lignin up to some extent^37^ (Fig. 4f). The cellulose obtained after the steam explosion (STEX) showed a peeling phenomenon due to the influence of the high-pressure steam used in the process, which led to the partial removal of waxes, pectin, and hemicellulose^38,39^. The destructive perforation of the lumen was also observed (Fig. 4g)^40^. It is in accordance with the compositional analysis data, where lignin content decreased from 21.1 to 2.3% and cellulose concentration increased from 39.2% in native rice straw to 85.5% in the A_12 sample. Based on the results, it was therefore concluded that the commercial process of cellulose extraction or the pre-treatment involving alkali is the most effective. Further, coupled with the bleaching process, which is most effective and causes the gradual discoloration of rice straw from pale brown to white^41^.

**Figure 5:**
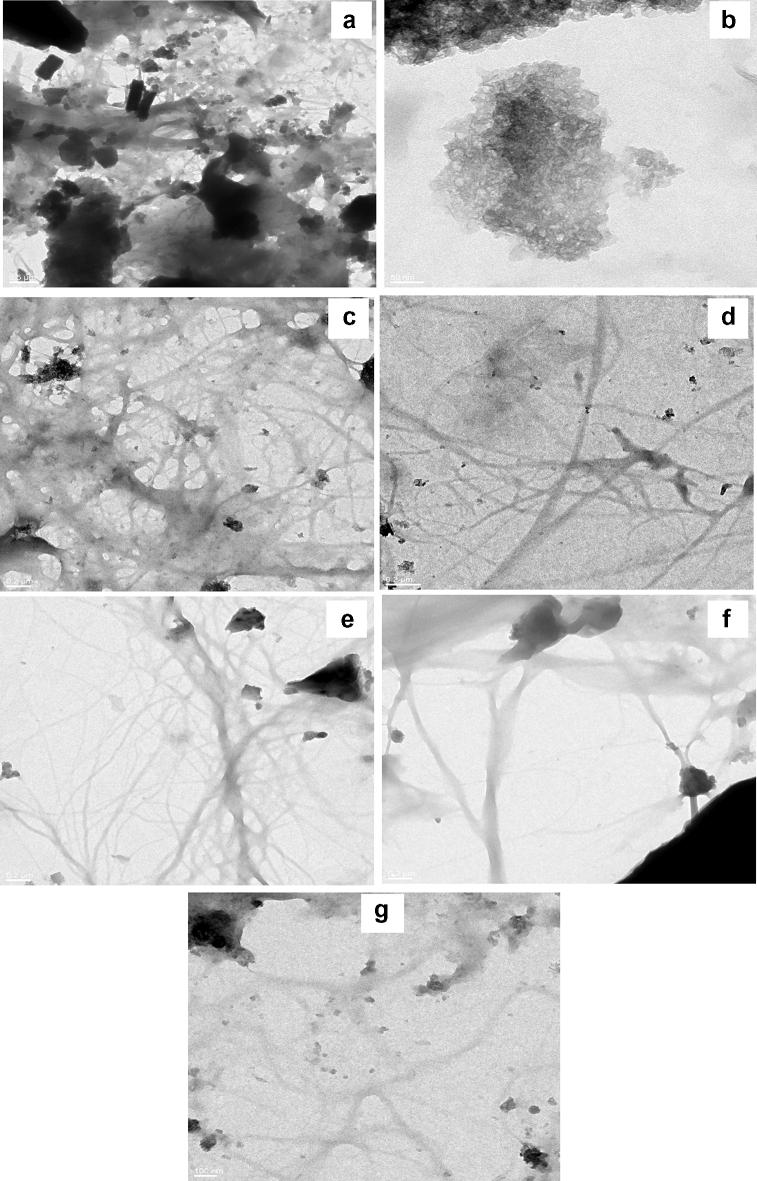
Transmission electron microscopy (TEM) images of treated samples (**a**) rice straw, (**b**) A_2, (**c**) A_5, (**d**) A_8, (**e**) A_12, (**f**) organosolv and (**g**) steam explosion.

The comparative investigation of alkali-treated cellulose samples and native rice straw through TEM showed rod-shaped cellulose fibres were visible in samples with higher concentrations of alkali (5% and above). As seen in the micrographs, A_2 showed clusters, which could be due to the unaltered intermolecular interactions between cellulosic and non-cellulosic components (Fig. 5a,b), while A_5, A_8, and A_12 indicated efficient removal of hemicellulose and lignin (Fig. 5c–e)^42^. As reported earlier, the rod-shaped fibres of cellulose were interlinked through a web-like network^42^. Similarly, fibrillated structures were also observed in STEX and organosolv-treated samples that confirmed the loosening of structure and breaking of intermolecular bonds, leading to improved accessibility of cellulosic fibrils, which is beneficial for various applications (Fig. 5f,g). Hence, the morphology of various pre-treated cellulosic samples indicated loosened cellulosic fibrillar structures.

### Chemical and thermal properties of native rice straw and extracted cellulose as determined by FTIR, XRD, and DSC

**Figure 6:**
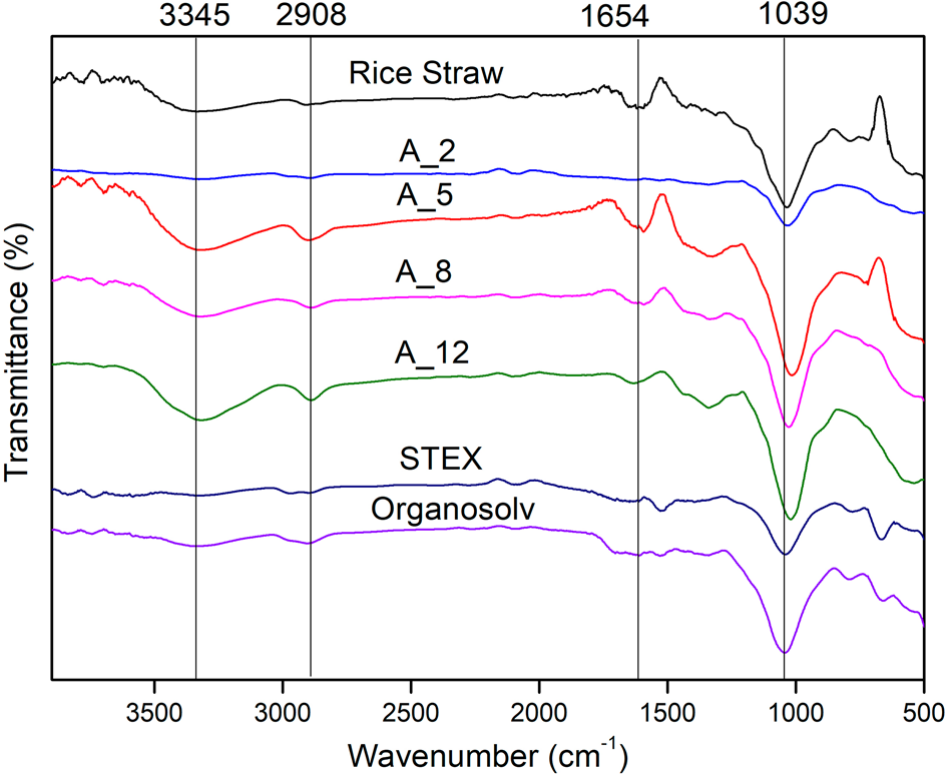
FTIR spectra of native rice straw and cellulose-rich variants obtained after various pre-treatment methods.

Rice straw is a heterogeneous complex of cellulose, hemicellulose, and lignin. FTIR spectroscopy was performed to study the changes in the chemical structures after various pre-treatments. FTIR spectra of rice straw and cellulose extracted using alkali, steam, and organosolvent treatment revealed the major absorptions corresponding to the cellulose and lignin functional groups (Fig. 6). It was observed that some of the components had been removed from the structure after the alkali, steam, and organosolvent treatments, as indicated by the disappearance of their respective peaks in the spectra. showed a broad peak at wavenumber 3345 cm^−1^ which corresponds to the O–H group stretching vibrations of the hydrogen-bonded hydroxyl groups observed in all of the rice straw and extracted cellulose variants^18,43^. Another intense peak at wavenumber 2908 cm^−1^ was found in polysaccharides representing the C-H bond, and a peak at 1654 cm^−1^ characteristically belonging to the vibration of C–O stretching of cellulose was also observed in all the variants^44^. The peaks at 1594 cm^−1^ and 1427 cm^−1^ were observed in native rice straw, representing the aromatic C–C stretch of aromatic vibration due to the bound lignin, but were not observed in the extracted cellulose variants, indicating the removal of lignin after various pre-treatments^40^. The absorption band at 1374 cm^−1^ which belongs to the C–H stretching vibrations due to the partial acetylation of hydroxyl groups in both the polysaccharides and the lignin, was present in the native rice straw but was absent in all the pre-treated samples, and hence inferring the delignification^45,46^. The band at wavenumber 1039 cm^−1^ which is assigned to the C–O–C pyranose ring of cellulose, was visible in all the treated samples (A_2, A_5, A_8, A_12, STEX, and organosolv)^47^. Thus, it is evident from the spectra that with the severity of the treatments employed for cellulose extraction, the peak intensity attributed to the lignin and other non-cellulosic components of fibres reduced significantly^48^.

**Figure 7:**
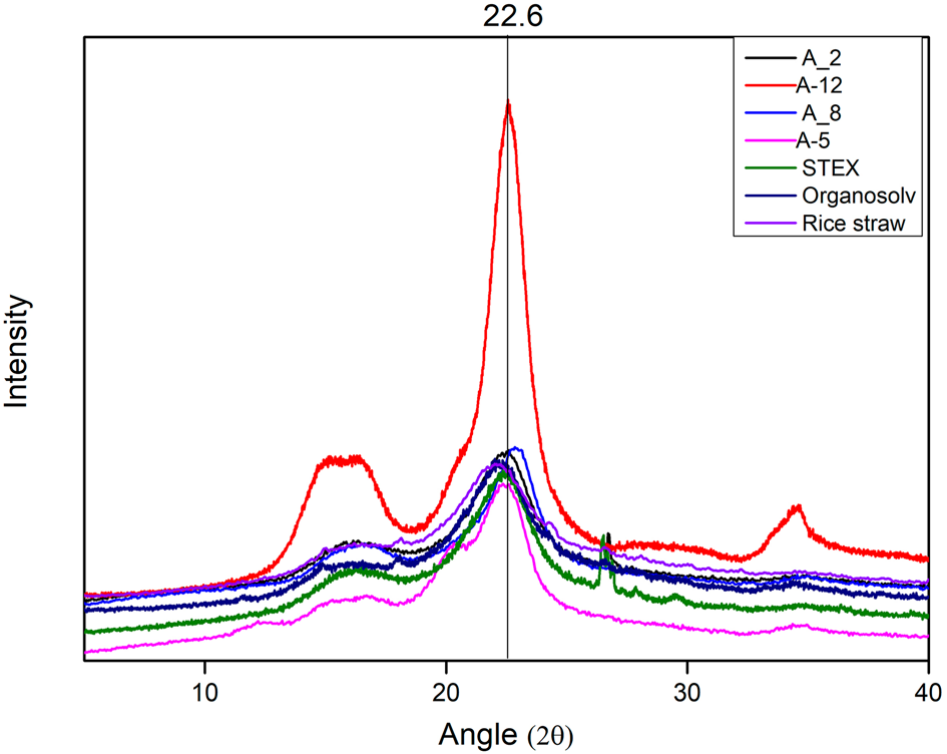
X-ray diffraction patterns of rice straw, A_2, A_5, A_8, A_12, steam explosion, and organosolv.

Alteration in the composition of cellulose, hemicellulose, or lignin affects the crystallinity index and may lead to a deviation in the typical XRD pattern^49^. To understand the impact of the adopted pre-treatment methodologies, XRD was performed for the native rice straw and the extracted cellulose variants (Fig. 7). All the cellulose samples showed lattice planes (1–10, 110, 004, 021, and 200) were observed in the cellulose of higher plants^50^. Compared to the native rice straw, an increase in the plane of 200 in the A_12 sample was observed due to the highly organized cellulose chain after alkali treatment. The CrI of native rice straw, A_2, A_5, A_8, A_12, STEX, and organosolv cellulose was 41.9%, 40.1%, 45.1%, 46.7, 52.2%, 48%, and 45%, respectively. An increase in CrI values in all samples has been attributed to (i) the organization of the crystallographic domains into a more ordered state and (ii) the removal of lignin and hemicellulose^51,52^. All the samples, especially A_12, showed variation in the XRD pattern and the CrI as compared to the native rice straw. Similar observations for an increase in CrI have been reported earlier for alkali pre-treatment of lignocellulose biomass^53^.

**Figure 8:**
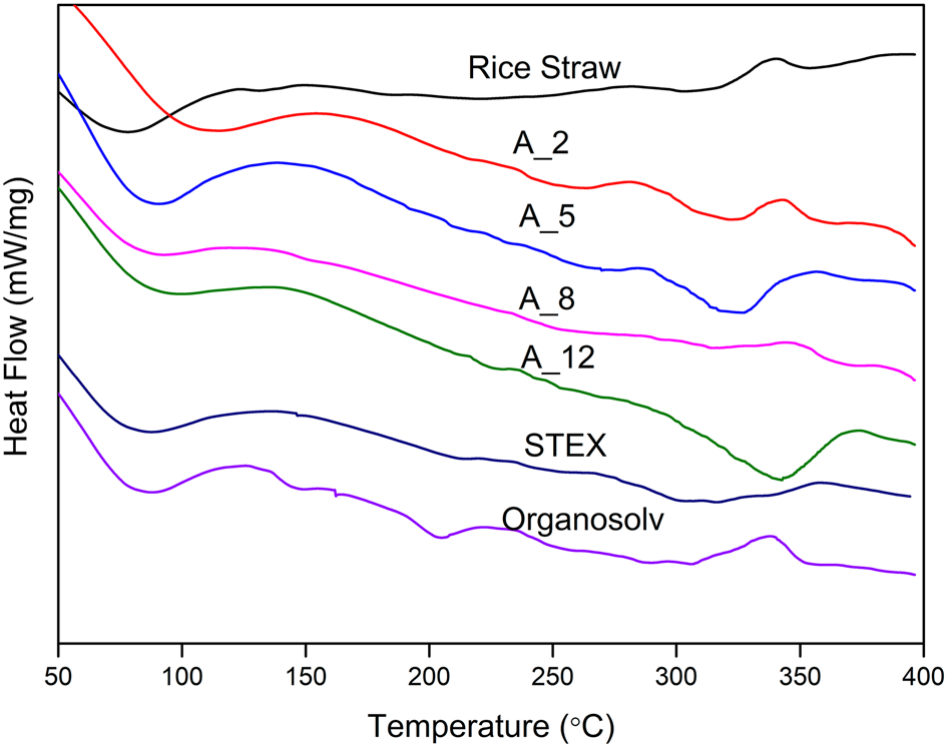
Differential Scattering Calorimetry (DSC) thermogram of native rice straw and cellulose samples.

To study the thermal behaviour of rice straw and extracted cellulose, differential scanning calorimetry (DSC) analysis was performed. The heat flow moving in or out of the extracted cellulose samples was measured as a function of temperature to evaluate their thermal stability. In Fig. 8, the DSC curve indicates the energy consumption properties of rice straw and the extracted cellulose samples. The DSC thermogram exhibited an early endothermic peak with an enthalpy change observed at 90–110 °C in the cellulose variants, whereas native rice straw showed a peak at 80 °C. This minor enthalpy change is attributed to the desorption of moisture as water trapped in the polysaccharide structure is released. The DSC profile of the organosolv-treated sample showed an endothermic peak at 203 °C due to non-cellulosic constituents such as hemicellulose^54^.

As the temperature increased further, the DSC profile of all the cellulose samples showed an endothermic peak at a range of 306–343 °C. However, A_12 showed the most prominent endothermic peak at around 343 °C. This could be due to the increase in compactness of the crystallinity structure caused by the breakdown of intra-molecular interaction and the decomposition of the cellulose^55^. Similar results were reported by Veeramachineni et al., where the endothermic peaks of the cellulose sample were in the range of 350–360 °C^56^.

### Cellulose nanofibres (CNFs) production through ball milling

Cellulose nanofibres (CNFs) were synthesized using ball milling, which is an environmentally friendly and cost-effective process^57^. The purest cellulose variant (A_12) was used for nanofibres production through ball milling. Ball milling provided a shear that helped in the conversion of cellulose fibres into nanofibres. The effectiveness of ball milling was studied based on the optimization of three critical factors: time (3 h), speed (300 rpm), and ball-to-material ratio (BMR-80:1). The ball-milled samples were stored under refrigerated conditions for further characterization.

### Morphological characterization of cellulose nanofibres using TEM and EDX

**Figure 9:**
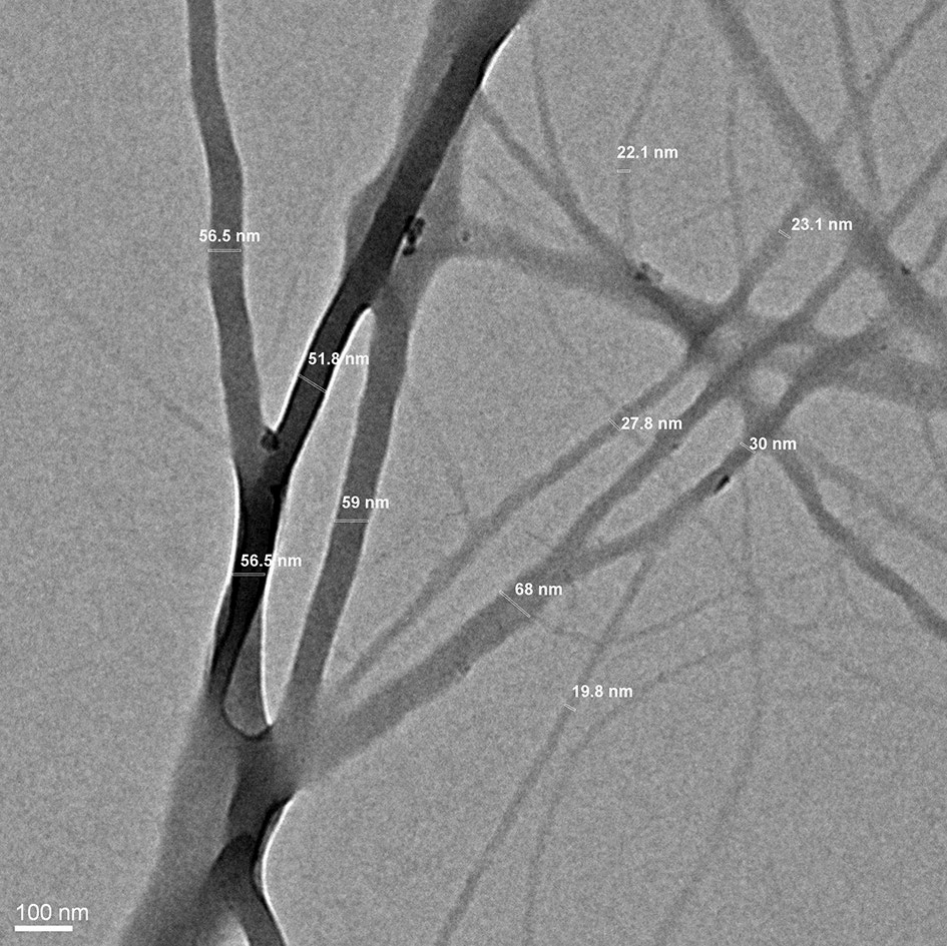
TEM micrograph of cellulose nanofibres.

Transmission electron microscopy (TEM) was employed to confirm the synthesis of cellulose nanofibres (CNFs) from the rice straw. Cellulose nanofibres with diameters in the nanometer range (19.8–68 nm) were observed, as shown in the micrographs (Fig. 9). A similar result was also reported by Nasri-Nasrabadi, where the CNF with 70–90 nm diameters was produced by the chemo-mechanical method^58^. However, considering that the ends of nanofibres could not be discerned and the fibrils were not straight, TEM was not found suitable for determining the length of the CNFs. It was also realized that the ball milling of the chemically treated fibres resulted in the defibrillation of cellulose nanofibres in the form of a segregated nanofibre network. It is believed that the energy released due to collisions, impacts, and attrition between the balls and the cellulose powder, the ball-to-material ratio, speed, and time are the most effective factors in extracting the nanofibres.

Energy dispersive X-ray analysis (EDX) was performed for the cellulose samples as it provides an estimate of the elemental composition of the materials. The ball-milled A_12 sample revealed the presence of carbon (C) and oxygen (O); the graph is given in Supplementary Fig. S1. A similar EDX result was reported where carbon and oxygen were found along with the sodium contamination that may occur because of the alkali pre-treatment^59^.

### FTIR spectroscopy, Zeta potential, and XRD of cellulose nanofibres

The FTIR spectrum of CNFs showed similar peaks as those found in the cellulose samples, viz*.,* the C–H bond corresponds to 2884 cm^−1^, C–O stretching of cellulose at 1637 cm^−1 ^and the C–O–C pyranose ring of cellulose at 1022 cm^−1^. Thus, it confirmed that no other functional groups were incorporated during the milling process. The surface charge is the key factor for determining the stability of nano-sized particles in colloidal suspension and could be determined by measuring the zeta potential. Based on the estimations, the zeta potential of cellulose nanofibres was found to be − 25.2 mV, an anionic charge indicated the presence of hydroxyl groups on the surface of the cellulose nanofibres^15^.

The XRD spectrum of cellulose nanofibres reflected a higher intensity peak due to the removal of the amorphous component during the pre-treatment. The percentage of CrI was increased to 65% after the mechanical fibrillation as compared to the parent A_12 cellulose, which had a CrI of 52.2%. The spectrum has been provided in the supplementary file (Supplementary Fig. S2). As reported by Amiralian et al., no significant variation in the crystalline structure of CNF was observed after ball milling^44^. In another report, the crystallinity index of ball milled kenaf fibres (120 min) was around 64%, which is comparable with the rice straw cellulose that was ball milled for 3 h^60^.

Thus, the results concluded that the isolated cellulose from rice straw is comparable with the commercially available cellulose based on its chemical structure, morphology, and thermal properties. While the report by Johar et al., where cellulose was extracted from the rice husk through chemical and bleaching treatments, confirmed the removal of non-cellulosic materials, the chemical treatments induced an increase in the crystallinity index from 46.8 to 59.0%^17^. The increased crystallinity index of cellulose and CNF imparts good tensile strength, which is important for various applications.

## Conclusion

The demand for utilizing stubbles like rice straw is growing as a solution to residues burning in open fields, thereby reducing air pollution. However, the cellulose and the cellulose nano-fibres (CNFs) are bio-based and biodegradable, which could make them a promising material to solve issues related to open field burning. Hence, cellulose nanofibers extracted from rice straw could be an alternative nanocarrier to deliver the agrochemicals. This study revealed that the A_12 sample, where 12% NaOH was used, showed the maximum yield and potential fibrillation in scanning micrographs. The percentage of crystallinity index increased from 52.2 to 65% after the milling of cellulose fibres ball milling process. The surface charge, surface area, and accessibility of functional groups were found to be optimal and very promising for the binding of agrochemicals and the utilization of cellulose nanofibers for the development of biodegradable slow-release fertilizers delivery systems or controlled fertilizers delivery systems. This approach would pave the way to finding scalable and alternative options to control the environmental menace by utilizing the valuable natural resources within agricultural biomasses.

### Future prospects

Cellulose nanofibres have attracted tremendous attention because of their excellent chemical and physical properties and their renewability and sustainability. Cellulose and cellulose nanofibres derived from rice straw can be considered for other alternative uses. Thus, these characterizations could only elucidate the potential for alternate uses and routes. However, future studies should aim to validate these results in the development of value-added products such as whose utilized in the paper industry, brick preparation, animal food blocks, etc.

### Supplementary Information

Supplementary Information.

## Data Availability

All data generated or analysed during this study are included in this published article [and its supplementary information files].
