# Supplementary material for: Rice straw-derived cellulose: a comparative study of various pre-treatment technologies and its conversion to nanofibres
Source: Sci Rep. 2023 Sep 28;13:16327. doi: 10.1038/s41598-023-43535-7 (PMC10539515; doi:10.1038/s41598-023-43535-7)
Supplement: Supplementary file 1 — Supplementary Information. [file 41598_2023_43535_MOESM1_ESM.docx]

**Supplementary file**

**Rice straw derived Cellulose: A comparative study of various pre-treatment technologies and its conversion to nanofibres**

**Experiment 1:** To determine the elemental contamination that may occur during ball milling process for converting cellulose to nanocellulose.

**Fig.S1** EDX graph of ball milled A_12

The intensity of carbon and oxygen was seen in the cellulose nanofibres sample. The cellulose nanofibres have the glucose backbone made up of carbon and oxygen. Thus, it confirms that ball milling doesn’t introduce any contaminants in the sample.

**Experiment 2:** To estimate the functional and crystallinity change in cellulose nanofibres developed through ball milling.


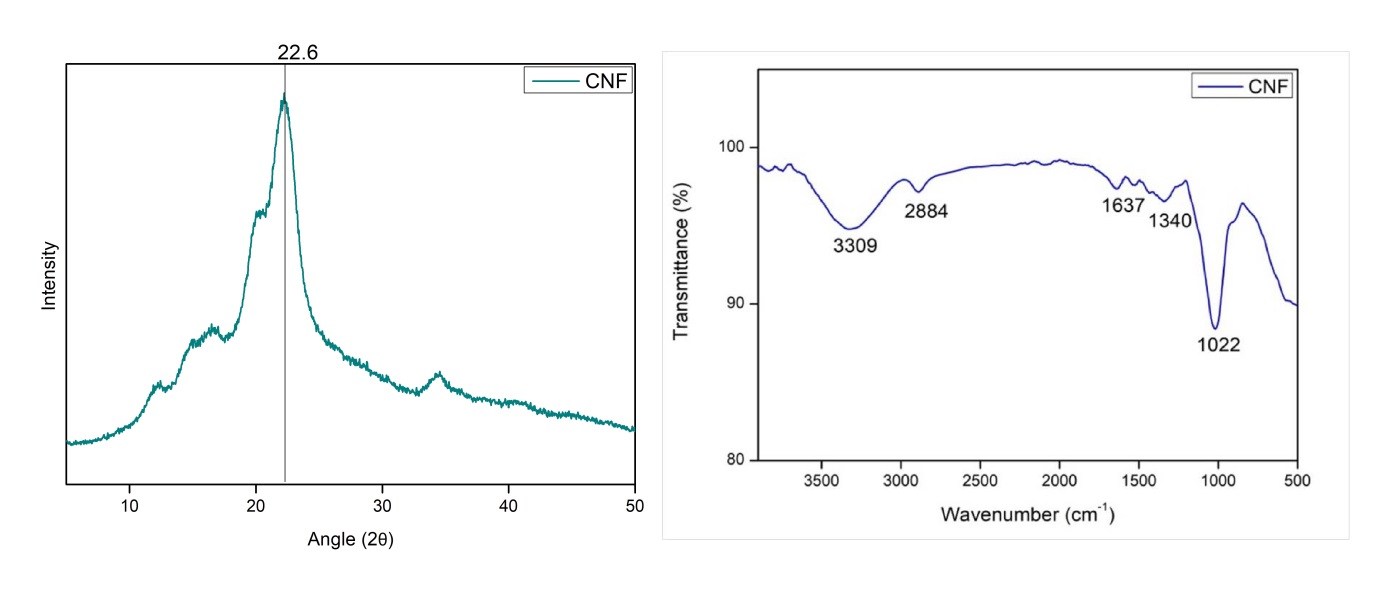


**Fig. S2.**  XRD and FTIR spectrum of cellulose nanofibres

The crystallinity index of cellulose nanofibres were around 65% and the FTIR spectrum showed no additional functional groups after mechanical milling. Thus, confirms the absence of metal contaminants.
